# Doubly Optimized Calibrated Support Vector Machine (DOC-SVM): An Algorithm for Joint Optimization of Discrimination and Calibration

**DOI:** 10.1371/journal.pone.0048823

**Published:** 2012-11-06

**Authors:** Xiaoqian Jiang, Aditya Menon, Shuang Wang, Jihoon Kim, Lucila Ohno-Machado

**Affiliations:** 1 Division of Biomedical Informatics, University California San Diego (UCSD), La Jolla, California, United States of America; 2 Deptartment of Computer Science and Engineering, University California San Diego (UCSD), La Jolla, California, United States of America,; National Cancer Institute, United States of America

## Abstract

Historically, probabilistic models for decision support have focused on discrimination, e.g., minimizing the ranking error of predicted outcomes. Unfortunately, these models ignore another important aspect, calibration, which indicates the magnitude of correctness of model predictions. Using discrimination and calibration simultaneously can be helpful for many clinical decisions. We investigated tradeoffs between these goals, and developed a unified maximum-margin method to handle them jointly. Our approach called, Doubly Optimized Calibrated Support Vector Machine (DOC-SVM), concurrently optimizes two loss functions: the ridge regression loss and the hinge loss. Experiments using three breast cancer gene-expression datasets (i.e., GSE2034, GSE2990, and Chanrion's datasets) showed that our model generated more calibrated outputs when compared to other state-of-the-art models like Support Vector Machine (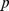 = 0.03, 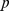 = 0.13, and 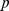
<0.001) and Logistic Regression (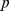 = 0.006, 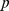 = 0.008, and 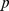
<0.001). DOC-SVM also demonstrated better discrimination (i.e., higher AUCs) when compared to Support Vector Machine (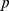 = 0.38, 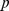 = 0.29, and 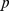 = 0.047) and Logistic Regression (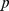 = 0.38, 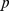 = 0.04, and 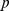
<0.0001). DOC-SVM produced a model that was better calibrated without sacrificing discrimination, and hence may be helpful in clinical decision making.

## Introduction

Supervised learning has been widely applied in bioinformatics [1]. Given sufficient observations and their class memberships, the prediction task is often modeled by supervised learning algorithms, which aim at finding an optimal mapping between features and outcomes (usually represented by the zero-one class membership). In clinical predictions, *discrimination* measures the ability of a model to separate patients with different outcomes (e.g., positive or negative). In the case of a binary outcome, good *discrimination* indicates an adequate distinction in the distributions of predicted scores. That is, *discrimination* is determined by the degree of correct ranking performance of predicted scores [2]. On the other hand, *calibration* reflects the level to which observed probabilities match the predicted scores [3], e.g., the prediction average is 60% for every individual in a group of observations and the proportion of the positive observations is also 60% in that group.

Traditionally, many machine learning models were developed to optimize discrimination ability [4], (i.e., minimizing the errors in making binary decisions based on the model's estimates). However, in many direct-to-consumer applications (i.e., using molecular biomarkers for diagnostic or prognostic purposes [5], [6]), estimated probabilities are being communicated directly to patients, hence calibration is very important. For example, clinicians may use estimated probabilities to make decisions related to prophylaxis for breast cancer. Achieving high levels of calibration in predictive models has become very important in clinical decision support and personalized medicine [7], [8], [9], [10], [11].”

**Figure 1:**
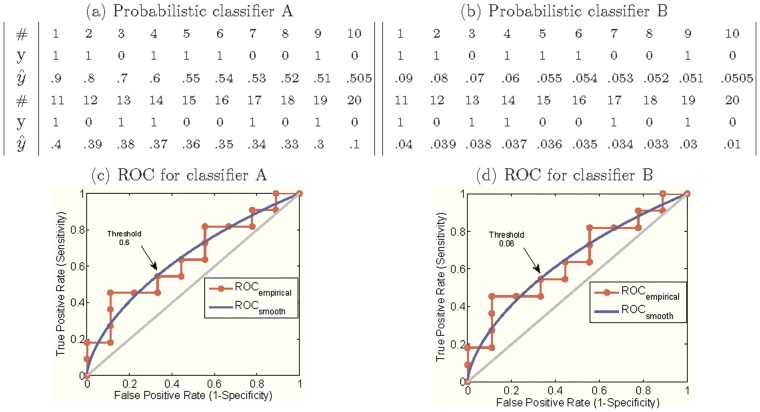
An example of outputs for two probabilistic classifiers and their ROC curves, which do not evaluate calibration. In (a) and (b), # indicates the observations, 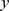 corresponds to the class membership, and 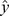 represents the probability estimate. In (c) and (d), each red circle corresponds to a threshold value. Note that probabilistic classifier B has the same ROC as probabilistic classifier A, but their calibration differs dramatically: estimates for B are ten times lower than estimates for A.

Good *discrimination* may lead to good *calibration*, but this is not guaranteed. A highly *discriminative* classifier, i.e., one with a large Area Under the ROC Curve (AUC), might not necessarily be a calibrated one. Figure 1 illustrates an example with 20 simulated subjects. While two probabilistic model A and B have the same AUCs, the values of probabilities from model B are ten times smaller than those from model A. Although *discrimination* estimates the ranking of subjects and their class membership, it does not account for the consistency between probabilistic model predictions and the true underlying probabilities. In extreme cases, a classifier can draw a perfect decision boundary but produces unrealistic risk estimates (e.g., by estimating a probability of “0.01” for negative observations and “0.011” for positive observations). Thus, significant problems may occur when direct outputs of supervised classification models are blindly used as proxies to evaluate the “true risks”.

In summary, although it is relatively easy to evaluate rank of estimates, it is non-trivial to convert these rankings into reliable probabilities of class membership, which is an important problem in personalized clinical decision making [12]. We want to find an accurate estimation of 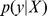: the probability that a subject 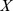 belongs to class 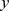, without sacrificing the *discriminative* ability of the model. Note that we used 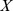 and 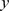 to denote the features and class label of an observation because the former represents a vector, while the latter refers to a scalar. In this article, we first investigate relationships between *discrimination* and *calibration*, then we proceed to show why it is beneficial to optimize *discrimination* and *calibration* simultaneously. We developed the Doubly Optimized Calibrated Support Vector Machine (DOC-SVM) algorithm that combines the optimization of *discrimination* and *calibration* in a way that can be controlled by the users. We evaluated our approach using real-world data and demonstrated performance advantages when we compared to widely used classification algorithms, i.e., Logistic Regression [13] and Support Vector Machine [14], [15].

## Methods

### Ethics Statement

We use two sets of breast cancer gene expression data with corresponding clinical data downloaded collected from the NCBI Gene Expression Omnibus,i.e., WANG (GSE2034) and SOTIRITOU (GSE2990), as well as another breast cancer gene expression data from Chanrion's group [16] in studying the occurrence of relapse as a response to tamoxifen. Because all these data are publicly available, we do not need IRB approval to use them.

### Preliminaries

We first review *discrimination* and *calibration* before introducing details of our methodology.

**Figure 2:**
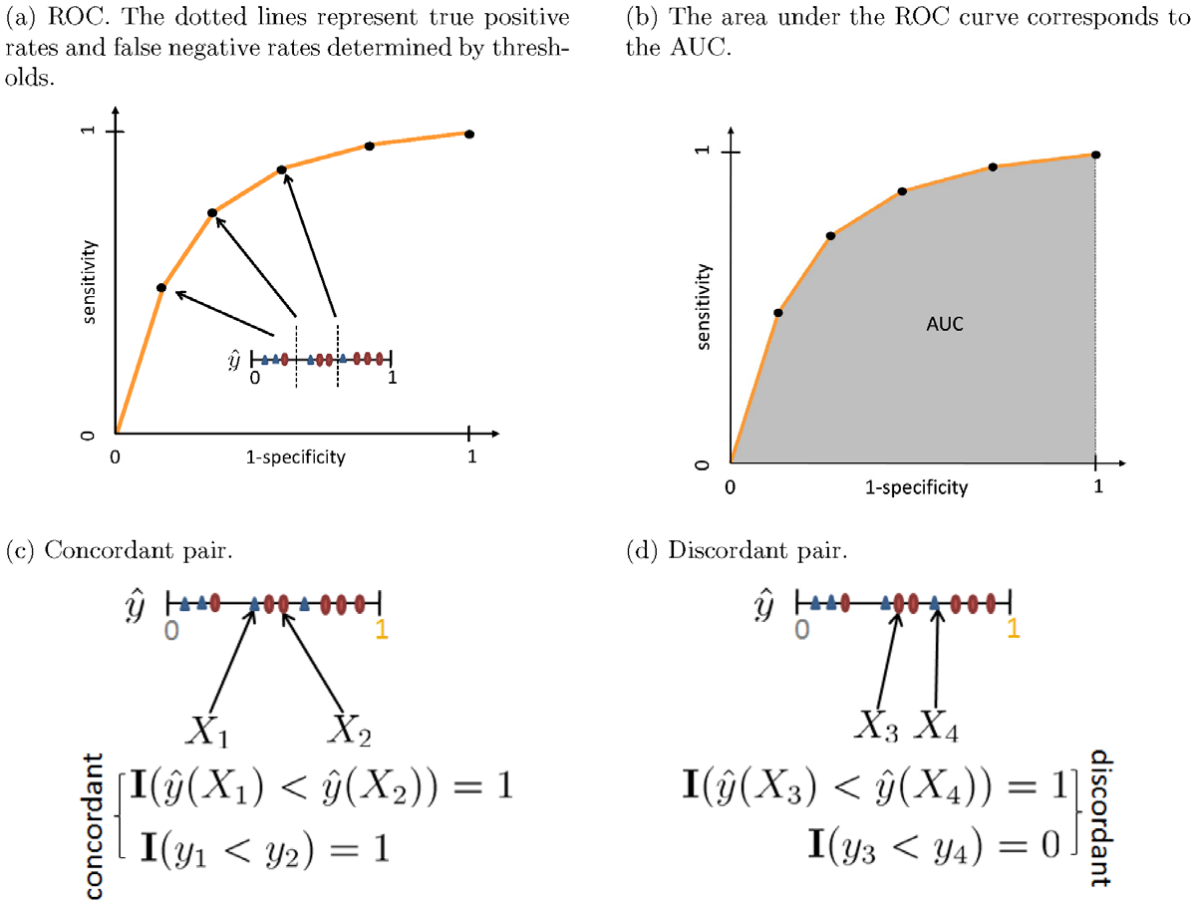
ROC, AUC and its calculation. The horizontal line shows sorted probabilistic estimates on “scores” 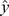
s. In (a) and (b), we show the ROC and the AUC for a classifier built from an artificial dataset. In (c) and (d), we show concordant and discordant pairs, where concordant means that an estimate for a positive observation is higher than an estimate for a negative one. The AUC can be interpreted in the same way as the c-index: the proportion of concordant pairs. Note that 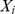 corresponds to an observation, 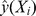 represents its predicted score, and 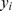 represents its observed class label, i.e., the gold standard. AUC is calculated as the fraction of concordant pairs out of a total number of instance pairs where an element is positive and the other is negative. Note that 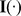 is the indicator function.

The Area Under ROC Curve (AUC) is often used as a *discrimination* measure of the quality of a probabilistic classifier, e.g., a random classifier like a coin toss has an AUC of 0.5; a perfect classifier has an AUC of 1. Every point on a ROC curve corresponds to a threshold that determines a unique pair of True Positive Rate (TPR = 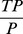) and False Positive Rate (FPR = 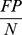), where 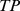, 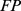, 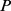 and 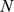 correspond to the number of true positive, false negative, positive, and negative observations, respectively. The AUC can be defined as the integral of TPR (also called *sensitivity*) over FPR (corresponds to *1-specificity*):
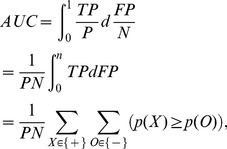
(1)where 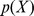 and 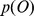 correspond to the estimates for a positive observation 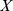 and a negative observation 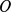, respectively. Note that 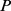 and 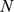 are the counts of positive and negative observations. The last line of Equation (1) corresponds to the result showed in [17] that AUCs can be seen as the total number of concordant pairs out of all positive and negative pairs, which is also known as the c-index [18]. For example, if all positive observations rank higher or the same as the negative observations, the AUC becomes 1; on the other hand, if none of the positive observations rank higher than any of the negative observations, the AUC value is 0. Figure 2 illustrates the relationships between ROC, AUC and its calculation. More details on parametric calculation of AUCs, please refer to [19].

**Figure 3:**
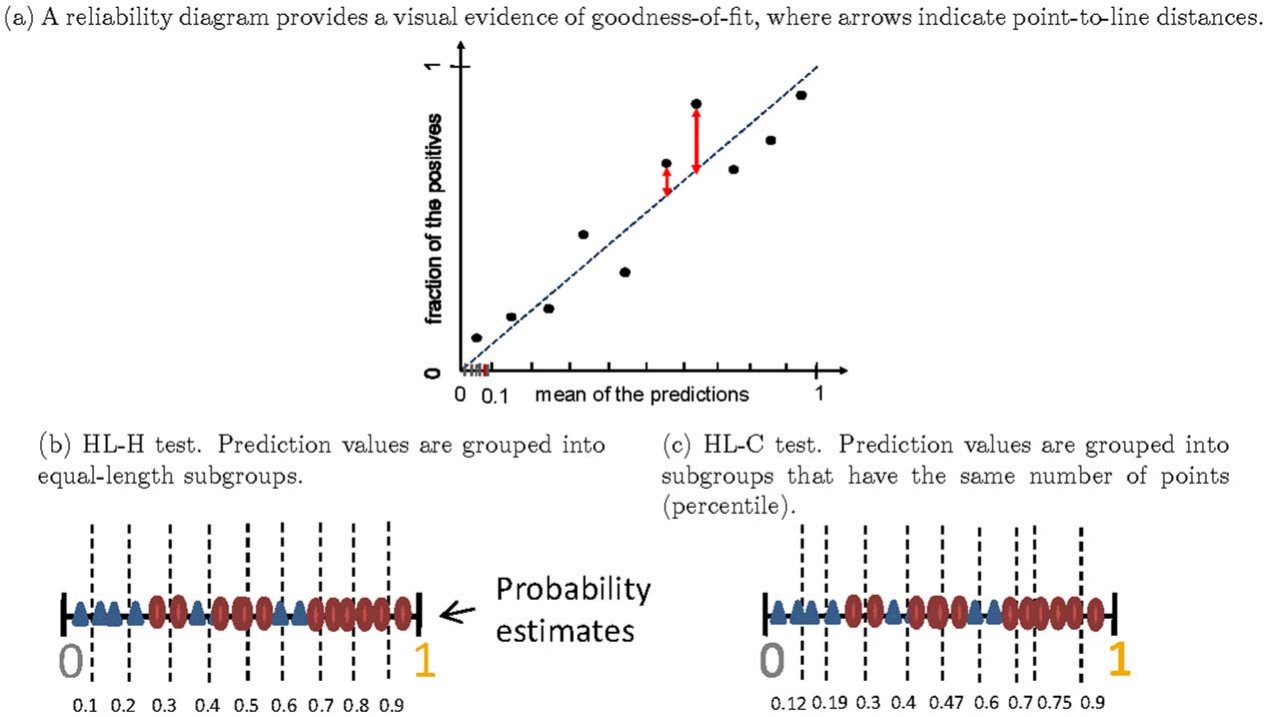
Reliability diagrams and two types of HL-test. In (a), (b), and (c), we visually illustrate the reliability diagram, and groupings used for the HL-H test and the HL-C test, respectively.

*Calibration* is a degree of agreement between predicted probability with actual risk, which can be used to evaluate whether a probabilistic classifier is reliable (i.e., faithful representative of the true probability). A probabilistic classifier assigns a probability 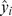 to each observation 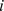. In a perfectly calibrated classifier, the estimated prediction 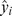 is equivalent to the percentage of positive events out of the population that receives this score (e.g., for a group of patients who receive a score of 0.25, one fourth will be positive for the outcome of interest, such as breast cancer). When there are few observations with the same probability, observations with similar probabilities are grouped by partitioning the range of predictions into groups (or bins). For instance, observations that were assigned estimates between 0.2 and 0.3 may be grouped into the same bin. To estimate the unknown true probabilities for many real problems, it is common to divide the prediction space into ten bins. Observations with predicted scores between 0 and 0.1 fall in the first bin, between 0.1 and 0.2 in the second bin, etc. For each bin, the mean of predicted scores is plotted against the fraction of positive observations. If the model is well calibrated, the points will fall near the diagonal line, as indicated in Figure 3(a).

*Calibration* can also be measured by goodness-of-fit test statistic, a discrepancy measure between the observed value form the data and the the expected values under the model under consideration. A widely used goodness-of-fit test in logistic regression is the Hosmer-Lemeshow test (HL-test) [20]. Although the HL-test has important limitations, few practical alternatives have been proposed. In addition, most of these alternatives are model-specific *calibration* measurements, which make them unattractive for evaluating probabilistic outputs (i.e., “scores”) across different models. For practical purpose, we use the HL-test as a measure of calibration in this article. The HL-test statistic can be written as 
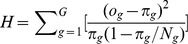
, where 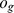, 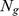 and 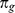 correspond to observed positive events, number of total observations, and the sum of predicted scores for the 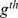 risk bin, respectively. 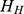 is the Hosmer-Lemeshow 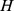 test statistic if a bin is defined by equal-length subgroups of fitted risk predictions, e.g., [0–0.1], [0.1–0.2], …, [0.9–1]; 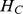 is the Hosmer-Lemeshow 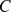 test statistic with an equal number of predicted scores in each group, e.g., 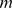 elements in group 1, 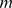 elements in group 2, …, 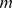 elements in the group 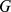. Usually, elements are divided into ten groups (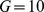), and the distribution of the statistics 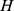 is approximated by a 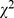 with 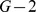 degrees of freedom, where 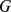 indicates the number of groups. Figure 3 illustrates a reliability diagram and two types of the HL-test. Note that in Figure 3(a), the small point-to-line distances roughly indicate that the model is reasonably *calibrated*, and it is not consistently optimistic or pessimistic.

#### Discrimination-Calibration Tradeoff

**Figure 4:**
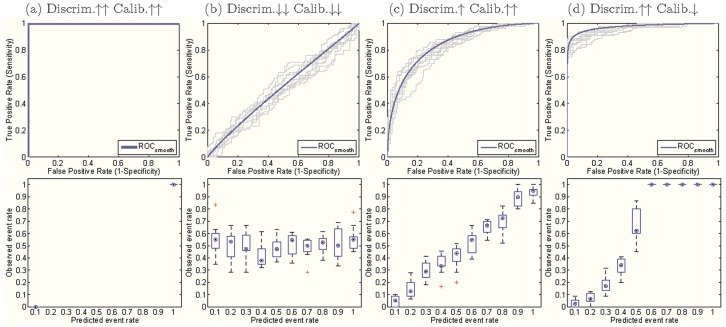
*Discrimination* plots (ROC curves) and *Calibration* plots for simulated models. (a) Perfect *discrimination* (i.e., AUC = 1) requires a classifier with perfect dichotomous predictions, which in the calibration plot has only one point (0,0) for negative observations and one point (1,1) for positive observations. (b) Poor *discrimination* (i.e., AUC = 0.53
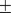
0.02) and poor *calibration* (i.e., 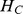 = 251.27
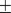
65.2, 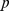
<1e−10). (c) Good *discrimination* (i.e., AUC = 0.83
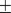
0.03) and excellent *calibration* (i.e., 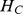 = 10.02
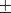
4.42, 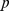 = 0.26
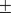
0.82). (d) Excellent *discrimination* (i.e., AUC = 0.96
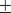
0.01) and mediocre *calibration* (i.e., 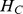 = 34.46
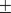
2.77, 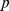 = 0
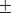
0.95). Note that a HL statistic smaller than 13.36 indicates that the model fits well at the significance level of 0.1.

Ideally, we want a model with good *discrimination* (i.e., 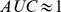) and good *calibration* (i.e., 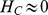). A perfect model occurs only when predictions are dichotomous (0 or 1) and predictions match observed class labels exactly. There are few conditions in which such black and white cases exit in the real-world. Even under such cases, the result might indicate the model overfits the training data. Figure 4(a) illustrates the situation of perfect *discrimination* and *calibration* in a training set. This usually does not guarantee the same behavior in the test set. Therefore, a realistic concern is whether *calibration* could be harmful to *discrimination*, and vice versa. In other words, suppose we construct a *calibrated* version of some classifier whose predictions are not dichotomous, could this increase the ranking error and hence decrease discrimination?

Figure 4(a, b, c, d) illustrates the relationship between *calibration* and *discrimination* with individual predictions 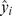, derived from a set of probabilistic models. Each subfigure illustrates ten models sampled at AUCs close to a given value (0.5, 0.8, and 0.95). In each column, the upper row represents the ROC plot, and the bottom row corresponds to the reliability diagram (i.e., *calibration plot*). In the ROC plot, grey curves denote empirical ROCs and the bold blue curve represents the averaged smooth ROC. In every *calibration* plot, we show the boxplot and histogram of observed event rates at predicted event rate intervals from 0.1 to 1. Note that a good *calibration* would be represented by boxplots that are roughly aligned with the 45 degree line.

**Figure 5:**
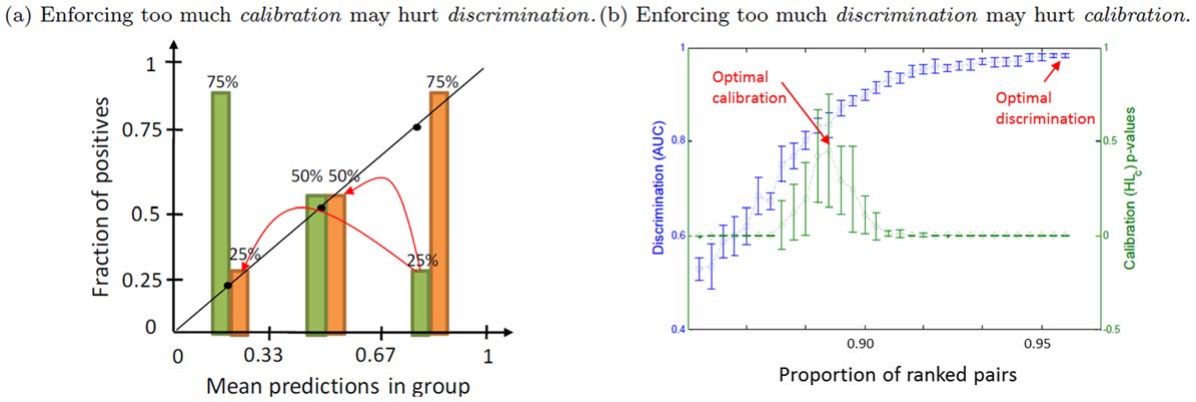
Tradeoffs between *calibration* and *discrimination*. (a) Perfect *calibration* may harm *discrimination* under a three-group binning. The numbers above each bar indicate the percentage of negative observations (green) and positive observations (orange) in each prediction group (0–0.33, 0.33–0.67, and 0.67–1). Note the small red arrows in the left figure indicate discordant pairs, in which negative observations ranked higher than positive observations. (b) Enforcing discrimination may also hurt calibration. The blue curve and error bars correspond to the AUC while the green curve and error bars represent the p-values for the Hosmer-Lemeshow C test (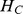). Initially, as *discrimination* increases, 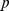-value of 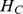 (*calibration*) increases but it quickly drops after hitting the global maximum. We use red arrows in Figure 5(b) to indicate the location of optimal calibration and discrimination for the simulated data.

The order of (b, d and c) shows that improvement in *calibration* on non-dichotomous predictions may lead to better *discrimination*, but further improvements in *calibration* might result in worse *discrimination*. The reason is that the perfect *calibration* for non-dichotomous predictions has to introduce discordant pairs (indicated by the red arrows in Figure 5(a)) to produce a match between the mean of predictions and the fraction of positive observations within each sub-group. Therefore, the model is prevented from being perfectly *discriminative*. This conclusion is concordant with the result of Diamond [21], who stated that the AUC of a perfectly non-trivially *calibrated* model (constructed from a unique, complementary pair of triangular beta distributions) cannot be over 0.83. Similarly, enforcing *discrimination* might hurt *calibration* as well. Figure 5(b) illustrates this situation using artificial data. Clearly, there is a tradeoff between *calibration* and *discrimination*, and we will explore it in more detail in the next section.

#### Joint Optimization Framework

We will show that *discrimination* and *calibration* are associated aspects of a well-fittedprobabilistic model, and therefore, they should be jointly optimized for better performance. We start introducing this global learning framework by reviewing the Brier score decomposition.

Brier Score Decomposition: The expectation of squared-losses between 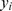 and 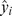 is also called *Brier score* [22]

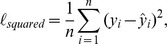
where 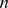 is the number of observations. Some algebraic manipulation leads to the following decomposition.

#### Lemma 1

*The Brier score can be expressed as*

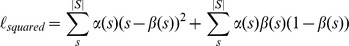
where 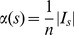, 
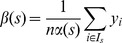
, and 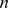 is the total number of observations, and 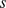 is a particular prediction value or score [23].

*Proof.* To prove that the Brier score can be decomposed into two components, we cluster predictions with the same estimated score 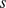. Thus 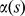 is the fraction of times that we predict the score 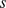, and 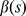 is the fraction of times that the event 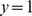 happens when we predict a score 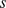. Note that 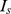 indicates a set of instances 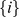 such that 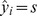, and 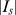 corresponds to the cardinality of the set.
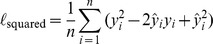


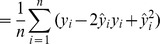


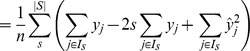


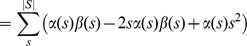


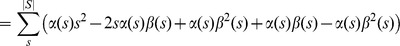


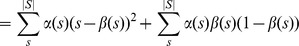



There are several versions of Brier score decompositions [24], [25], [26], [27], but for the interest of this article, we will focus on the above two-component decomposition. The first term of Brier score corresponds to *dis-calibration* (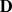) and its minimization encourages 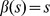, which is the exact condition required for well-*calibrated* estimations. The minimization of the second term, called *refinement* [28], encourages 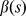 to be confident (i.e., close to 0 or 1). The *refinement* term (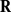), which indicates the homogeneity of predicted scores, is closely related to *discrimination*.

#### The Refinement Term

*Refinement* is a measure of *discrimination* but is often overlooked in favor of its sibling, AUC. Here, we study its properties more closely.

#### Lemma 2

*We can re-express refinement as*

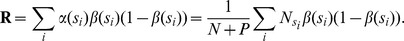



Note that 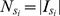 indicates the number of examples with the predicted score 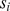, while 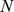 and 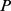 correspond to the number of negative and positive examples, respectively.

#### Lemma 3

*We can re-express the AUC calculated by the trapezoidal method* [*29*] *as*

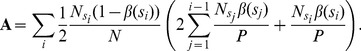
(2)


*Proof.* Each point of the ROC curve has width and height:
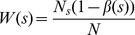


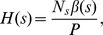
thus, the AUC can be approximated by summing over the trapezoidal areas under it:
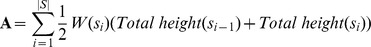


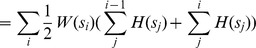


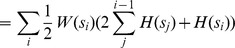


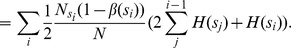



#### Theorem 4

*AUC is lower bounded by refinement:* 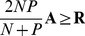.

*Proof.* We can reorganize Equation (2) as
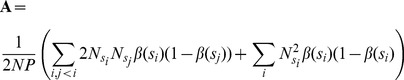
(3)


Because 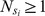,
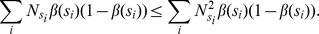
Thus, if we multiply 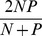 to both sides of Equation (3) and reorganize it,
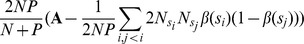


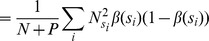


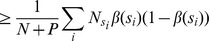


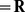



Since 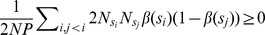, we showed that 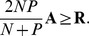


Theorem 4 indicates that maximizing *refinement* encourages the maximization of AUC, which is a critical result for combining *calibration* and *discrimination* into a unified framework.

#### Doubly Optimized Calibrated Support Vector Machine

We developed a novel approach called Doubly Optimized Calibrated Support Vector Machine (DOC-SVM) to jointly optimize *discrimination* and *calibration*. We will quickly review SVM to help understand the notation we use to explain DOC-SVM. Consider a training dataset 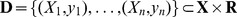, where 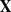 denotes the space of input patterns (e.g. 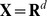) and class labels 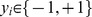. Here “+1” indicates a positive case and “−1” indicates a negative case. A Support Vector Machine (SVM) [15] approximates the zero-one loss by maximizing the geometric margin 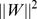 between two classes of data. The function it optimizes can be written as
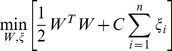
(4)

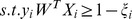


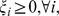
where 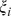 is the loss for the 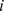-th data point 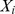; 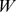 are weight parameters; and 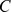 is a penalty parameter to weight the loss. We can reorganize Equation (3) by absorbing the constraints into the objective function
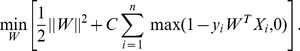
(5)


The first term 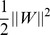 is responsible for the model's complexity. The second term 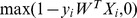, known as the hinge loss 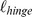, penalizes the model for mis-classifications. SVM expects label “1” cases to be 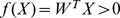 and label “−1” cases to be 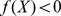. The final output of this optimization is a vector of weight parameters, 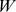, which forms a decision boundary that maximizes the margin between positive and negative cases.

As the hinge loss function only deals with decision boundary, SVM suffices in tasks where the mission is to provide good *calibration* besides *discrimination*. Some researchers proposed ad-hoc post-processing steps like Platt scaling [30] or Isotonic Regression [31] to rectify its output. Our idea is to introduce a second term, the squared loss, to be optimized concurrently with the hinge loss of the original SVM. As we discussed before, the squared loss (Brier Score) can be decomposed into *calibration* and *refinement* components. The major challenge for explicitly controlling the joint optimization is to integrate the *refinement* component with the *hinge loss* component to get a unified *discrimination* component. As we already know there is a relationship between *refinement* and AUC, the challenge boils down to identifying the relationship between the *hinge loss* and the AUC. There are some related empirical studies by Steck and Wang showing that the minimization of the hinge loss leads to the maximization of the AUC [32], [33] but we are the first to give a formal proof. Our proof is an extension of Lemma 3.1 in [34].

#### Theorem 5

*Rank loss (i.e., one minus the Area Under the ROC curve) is bounded by the hinge loss as* 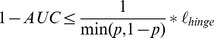 *, where* 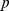 *is the probability of the positive class.*

*Proof.* Given a classifier 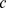 and 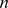 observed events 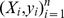, we can build the confusion matrix in Table 1, where TP, FP, FN, and TN denote the counts of true positive, false positive, false negative, and true negative instances, respectively.

**Table 1:** Confusion matrix of a classifier 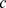 based on the gold standard of class labels.

		“Gold standard”	
		Positive	Negative
Predictions	Predicted positive	True Positive (FP)	**False Positive (FP)**
	Predicted negative	**False Negative (FN)**	True Negative (TN)

The number of maximum discordant pairs 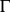 is bounded by
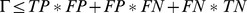


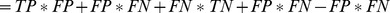


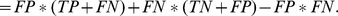
Dividing both sides by 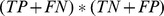, we get
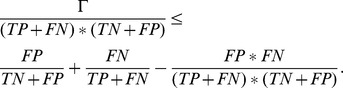
We can normalize 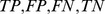 by the total number of records to get 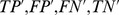 and their replacement of the formers to the above equation will not change the inequality. We can simplify the equation to get
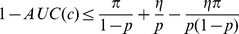
where 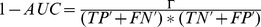, 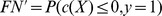 (denoted as 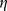), 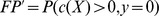 (denoted as 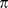), 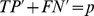 as the probability of the positive class, and 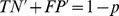 as the probability of the negative class. Therefore, as in Theorem 3.1 of Kotlowski [34],
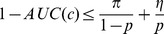


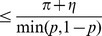


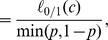
where 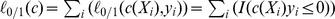 indicates the zero-one loss, 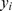 and 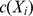 are the class label and the prediction score of the 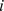-th element, and 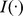 is an indicator function. Since the hinge loss function 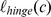 upper bounds the zero-one loss 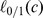 for an arbitrary classifier 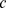 (i.e., 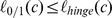), we proved that 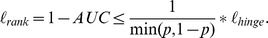


Although it provides a loose bound, Theorem 5 indicates that minimizing the hinge loss function leads to AUC maximization because 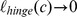 implies 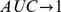. The following objective function optimizes the Doubly Optimized Calibrated Support Vector Machine (DOC-SVM),
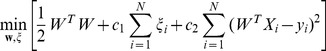
(6)

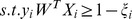


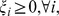
where 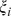 is the loss for the 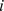-th data point 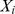; 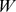 is the weight parameter; 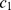 and 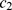 are the penalty parameters for the hinge loss the squared loss, respectively. DOC-SVM optimizes *discrimination* and *calibration* in a joint manner. Let us denote the hinge loss as 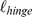, the squared loss as 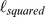, *refinement* as 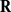, *dis-calibration* as 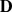, and AUC as 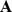. Holding the regularization term 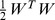 as a constant, Equation (6) concurrently optimizes *discrimination* and *calibration*, and it allows the explicit adjustment of the tradeoff between the two giving,
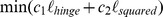


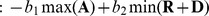


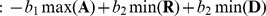


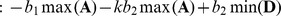


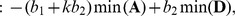
where 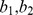 are weight parameters for different loss functions and 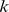 is a constant factor. As 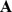 is lower bounded by a factor of 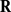, therefore, the minimization of 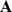 enforces the minimization of 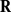. The higher 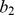 is, the less *discriminative* and the more *calibrated* the classifier is, and vice versa.

## Experiments

We evaluated the efficacy of DOC-SVM using three real-world datasets. We examined the *calibration* and *discrimination* of the LR, SVM, and DOC-SVM. To allow for a fair comparison, we applied Platt's method to transform SVM's outputs into probabilities [30]. We adopted ten-fold cross validation [35] to pick the best parameters (i.e., 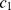, 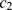 for DOC-SVM and 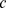 for SVM) for each model. Specially, we used the following metrics for evaluation: AUC, AUC standard deviation, F-score, Sensitivity, Specificity, Brier Score, and the p-value of the HL-C test, which are all among the most commonly used in statistical model comparisons. The null hypothesis in our HL-C test is that the data are generated by the model fitted by the researcher. If test statistic is less or equal to 0.1, we reject the null hypothesis that there is no difference between the observed and model-predicted values, which implies that the model's estimates do not fit the data well (i.e., the calibration is poor). Otherwise, if the test statistic is greater than 0.1, as expected for well-fitting models, we fail to reject the null hypothesis.

Our first experiment used breast cancer gene expression data collected from the NCBI Gene Expression Omnibus (GEO). Two individual datasets were downloaded, and were previously studied by Wang et al. (GSE2034) [36] and Sotiriou et al. (GSE2990) [37]. Table 2 summarized the data sets. To make our data comparable to the previous studies, we followed the criteria outlined by Osl et al. [38] to select patients who did not receive any treatment and had negative lymph node status. Among these pre-selected candidates, only patients with extreme outcomes, either poor outcomes (recurrence or metastasis within five years) or very good outcomes (neither recurrence nor metastasis within eight years) were selected. The number of observations after filtering was: 209 for GSE2034 (114 good/95 poor) and 90 for GSE2990 (60 good/30 poor). All of these data have a feature size of 247,965, which corresponds to the gene expression results obtained from certain micro-array experiments. Both datasets have been preprocessed to keep only the top 15 features by T-test, as previously described [38].

**Table 2:** Details of the training and test datasets in our first experiment.

	#ATTR	TRAINING SET SIZE	TEST SET SIZE	%POS
GSE2034	15	125	84	54%
GSE2990	15	54	36	67%

**Figure 6:**
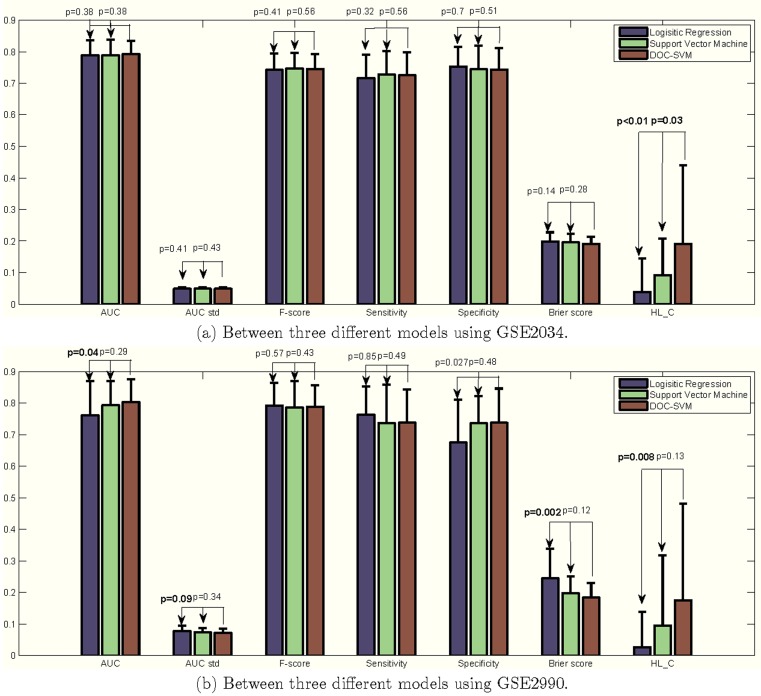
Performance comparison between using GSE2034 and GSE2990.

Figure 6(a) and Figure 6(b) illustrate a number of comparisons between LR, SVM, and DOC-SVM using the GSE2034 and GSE2990 datasets over 30 random splits. In both experiments, DOC-SVM showed higher AUCs when compared to other models under one-tailed paired t-tests using 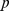 = 0.1 as the threshold. Although the improvements to SVM are small (GSE2034: 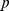 = 0.38, GSE2990: 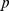 = 0.29), DOC-SVM had significantly higher AUCs compared to LR in GSE2990 (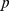 = 0.04). Besides discrimination, DOC-SVM demonstrated better calibration in terms of HL-C test. In the experiment, DOC-SVM had significantly higher 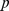-values than the LR model (GSE2034: 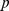
<0.01, GSE2990: 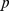 = 0.008) using a one-tailed paired t-test. An improvement to SVM was significant for GSE2034 (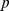 = 0.03) but not for GSE2990 (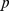 = 0.13). We also conducted one-tailed paired t-tests to evaluate if DOC-SVM has smaller Brier scores when compared to LR and SVM. The results were similar to what we already observed in discrimination and calibration: the Brier scores were smaller than those of LR (GSE2034: 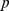 = 0.14, GSE2990: 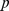 = 0.002) and SVM (GSE2034: 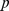 = 0.28, GSE2990: 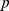 = 0.12), but not all improvements were significant. In no instances DOC-SVM performed significantly worse than SVM and LR.

Our second experiment used another breast cancer gene expression data, in which Chanrion and his colleagues predicted the occurrence of relapse as a response to tamoxifen [16]. We followed their experimental design, and conducted log2-transformation and median-centering per sample on the measurement values. To ensure consistency, we selected 36 genes present in their study and applied nearest shrunken centroid classification method [39]. Note that we carefully split the 155 observations into training and test sets to match what has been reported in that study. Data sets used are shown in Table 3.

**Table 3:** Details of the training and test datasets in our second experiment.

	#ATTR	DATASET SIZE	%POS
Training	36	132	65%
Test	36	23	74%

**Figure 7:**
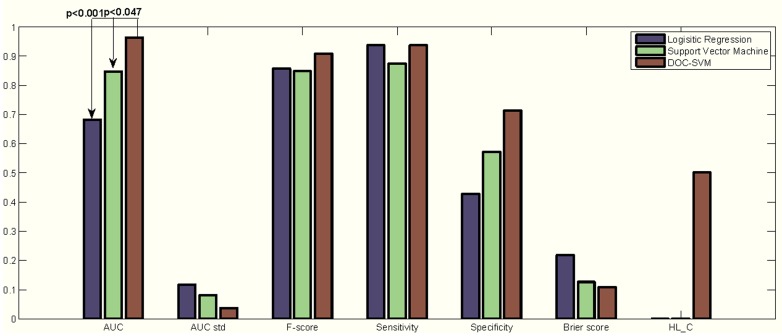
Performance comparisons between three different models using breast cancer datasets.

The evaluation of this experiment does not involve random split as the training and test datasets were predetermined [16]. Figure 7 shows indices for all three models.

DOC-SVM demonstrated better discrimination performance on the test data (AUC = 0.964), which was significantly higher than the AUCs of SVM (0.848) and LR (0.683). Note that for the comparison of a pair of AUCs, we used a z-test reviewed in Lasko et al. [40]. DOC-SVM also had the lowest Brier score among the three models. In addition, it was the only model that had a good fit, with a HL-C test 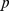-value equals 0.5, whereas 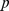-values of the other models were smaller than 0.0001.

In summary, DOC-SVM showed superior performance in all these real-world datasets. The performance improvements were observed for both *discrimination* and *calibration*, which indicates that DOC-SVM may have better generalization ability compared to LR and SVM due to the joint consideration of both factors. Although these experiments are limited by the small sizes of datasets, their outputs verified our theoretical results and served to demonstrate the advantage of the proposed joint optimization framework.

## Conclusions

We explored the properties of *discrimination* and *calibration*, and uncovered an important tradeoff between them, expressed in terms of AUC, a popular measure of *discrimination*. Our investigation also indicated that a supervised probabilistic model can be improved when both *discrimination* and *calibration* are considered in a joint manner. We developed a Doubly Optimized Calibrated Support Vector Machine Model (DOC-SVM) to minimize the squared loss concurrently with the hinge loss to account for both aspects of *discrimination* and *calibration*. Experimental results from using real-world breast cancer datasets indicate that the DOC-SVM can potentially outperform Logistic Regression and Support Vector Machine. Further studies are needed to investigate strategies to tune weights for *discrimination* and *calibration* depending on the learning problem.

## Supporting Information

Appendix S1 **subgradient descent optimization for SVM.**
(DOCX)

Appendix S2 **parameter tuning using ten-fold cross validation.**
(DOCX)

Appendix S3 **additional experiments.**
(DOCX)

Appendix S4 **DOC-SVM Matlab code.**
(DOCX)
