# Supplementary material for: Doubly Optimized Calibrated Support Vector Machine (DOC-SVM): An Algorithm for Joint Optimization of Discrimination and Calibration
Source: PLoS One. 2012 Nov 6;7(11):e48823. doi: 10.1371/journal.pone.0048823 (PMC3490990; doi:10.1371/journal.pone.0048823)
Supplement: Appendix S1 — subgradient descent optimization for SVM. (DOCX) [file pone.0048823.s001.docx]

**Appendix s1**

We introduce a simple optimization procedure for the support vector machine as described in Equation (5). Recall that objective is

$$G\left( W \right)=\min_{W} \left[ \frac{1}{2}\left| \left| W \right| \right|^{2}+C\sum_{i=1}^{n} \max\left( 1-y_{i}W^{T}X_{i},0 \right) \right]$$

Because it is not strictly monotonic, we can calculate the subgradient directions of the objective function as follows,

$$\frac{\partial G}{\partial W}=W+\sum_{i} \left\{ \begin{aligned} \begin{matrix} 0 & -y_{i}W^{T}X_{i}<-1 \end{matrix} \\ \begin{matrix} -\frac{y_{i}X_{i}}{2} & y_{i}W^{T}X_{i}=1 \end{matrix} \\ \begin{matrix} -y_{i}X_{i} & -y_{i}W^{T}X_{i}>-1 \end{matrix} \end{aligned} \right.$$

and iteratively optimize the support vector machine objective.

**Algorithm 1:** Subgradient descent optimization for SVM

Input: Features $\boldsymbol{X}$, labels $\boldsymbol{y}$, parameter $C$, precision $\epsilon$

Output: Learned weight parameters $W$

1. Initilaize weight parameters $W$ with random values ranging from 0 to 1.
2. For all elements $X_{i}\in\boldsymbol{X}$**,** $y_{i}\in\boldsymbol{y}$ calculate $\Delta W=\frac{\partial G}{\partial W}$ using the equation above the algorithm.
3. Update $W_{t+1}=W_{t}+\eta\Delta W$, where $t$ is an index for the iteration and $\eta$ is a step size parameter (i.e., a small number like $0.01$)
4. If the maximum difference between $W_{t+1}$ and $W_{t}$ is smaller than the precision $\epsilon$, terminate the algorithm and return outputs. Otherwise, iterate step 2-3 until convergence.
