# Supplementary material for: Doubly Optimized Calibrated Support Vector Machine (DOC-SVM): An Algorithm for Joint Optimization of Discrimination and Calibration
Source: PLoS One. 2012 Nov 6;7(11):e48823. doi: 10.1371/journal.pone.0048823 (PMC3490990; doi:10.1371/journal.pone.0048823)
Supplement: Appendix S2 — parameter tuning using ten-fold cross validation. (DOCX) [file pone.0048823.s002.docx]

**Appendix s2**

For SVM and DOC-SVM models, we determined parameters (*c1, c2, C*) using ten-fold cross validation by sweeping the range of $({10}^{-4},{10}^{-3},{10}^{-2},\ldots,{10}^{2},{10}^{3},{10}^{4})$. Because DOC-SVM has two parameters, we searched all combinations of *c1, c2* values.

Table 1: Selected parameters using ten-fold cross validation for three datasets

|  | *c1* | *c2* | *C* |
| --- | --- | --- | --- |
| GSE2034 | 0.001 | 1 | 0.1 |
| GSE2990 | 0.1 | 1 | 0.01 |
| Chanrion’s data | 10 | 10 | 0.01 |
