# Supplementary material for: Doubly Optimized Calibrated Support Vector Machine (DOC-SVM): An Algorithm for Joint Optimization of Discrimination and Calibration
Source: PLoS One. 2012 Nov 6;7(11):e48823. doi: 10.1371/journal.pone.0048823 (PMC3490990; doi:10.1371/journal.pone.0048823)
Supplement: Appendix S3 — additional experiments. (DOCX) [file pone.0048823.s003.docx]

**Appendix s3**

We also conducted additional experiments using Isotonic regression to calibrate SVM outputs. Because Isotonic regression (like Platt scaling) is a monotonic post-processing of the probabilities, the results for AUC, F-score, sensitivity and specificity do not change. There are some small differences of Brier score and HL-C (calibration p-value) compared to those obtained using the Platt scaling mechanism, as illustrated in the following figures.


(a) Performance comparision for three different models using GSE2034.


(b) Performance comparision for three different models using GSE2990.


(c) Performance comparision for three different models using breast cancer data.

For example, in the case of GSE 2034, the performance (Brier score and HL-C) of using Isotonic regression is slightly worse than that of Platt scaling. But the performance of using isotonic regression for GSE2990 and the breast cancer data is better than that of the Platt scaling. Regarding the performance comparision for SVM and DOC-SVM, there are no **significant** differences between using Platt scaling and using Isotonic regression to post-process SVM ouputs.
