# Supplementary material for: Doubly Optimized Calibrated Support Vector Machine (DOC-SVM): An Algorithm for Joint Optimization of Discrimination and Calibration
Source: PLoS One. 2012 Nov 6;7(11):e48823. doi: 10.1371/journal.pone.0048823 (PMC3490990; doi:10.1371/journal.pone.0048823)
Supplement: Appendix S4 — DOC-SVM Matlab code. (DOCX) [file pone.0048823.s004.docx]

**Appendix s4**

function [W,b]=SVM(N,M,XP,XN,C)

% The goal is to find a function f(x) = W'*x - b that classifies the non-

% separable points {xp_1,...,xp_N} and {xn_1,...,xn_M} by seeking a trade-off

% between the number of misclassifications and the width of the separating

% slab. Note that xp, xn, N and M indciate a negative sample, a positive sample, the size of negative samples and the size of positive samples, respectively.

% Model parameters W and b can be obtained by solving the following problem:

% minimize ||W||_2 + C*(1'*u + 1'*v)

% s.t. W'*xp_i - b >= 1 - u_i for i = 1,...,N

% W'*xn_i - b <= -(1 - v_i) for i = 1,...,M

% u >= 0 and v >= 0

% where C gives the relative weights of the number of misclassified

% points compared to the width of the slab.

% Solution via CVX

cvx_begin

variables W(n) b(1) u(N) v(M)

minimize (norm(W) + C *(ones(1,N)*u + ones(1,M)*v) )

XP'*W - b >= (1 - u);

XN'*W - b <= -(1 - v);

u >= 0;

v >= 0;

cvx_end

end

function [W,b]=DOC_SVM(N,M,XP,XN,c_1,c_2)

% The goal is to find a function f(x) = W'*x - b that classifies the non-

% separable points {xp_1,...,xp_N} and {xn_1,...,xn_M} by seeking a trade-off

% between the number of misclassifications and the width of the separating

% slab. Note that xp, xn, N and M indciate a negative sample, a positive sample,

% the size of negative samples and the size of positive samples, respectively.

% Model parameters W and b can be obtained by solving the following problem:

% minimize ||W||_2 + c_1*(1'*u + 1'*v) + c_2* ||W'*X -b -Y||_2

% s.t. W'*xp_i - b >= 1 - u_i for i = 1,...,N

% W'*xn_i - b <= -(1 - v_i) for i = 1,...,M

% u >= 0 and v >= 0

% where C gives the relative weights of the number of misclassified

% points compared to the width of the slab.

% Note that X ={xp_1,...xp_N,xn_1,...,xn_M} and Y=[ones(N,1);-ones(M,1)]

% correspond to combined feature and labels from both positive and

% negative classes.

% Solution via CVX

Y = [ones(N,1);-ones(M,1)];

cvx_begin

variables W(n) b(1) u(N) v(M)

minimize (norm(W) + c_1 *(ones(1,N)*u + ones(1,M)*v) + c_2*norm([XP; XN]'*W-b - Y ) )

XP'*W - b >= (1 - u);

XN'*W - b <= -(1 - v);

u >= 0;

v >= 0;

cvx_end

end
